# 

**Published:** 1912-01

**Authors:** 


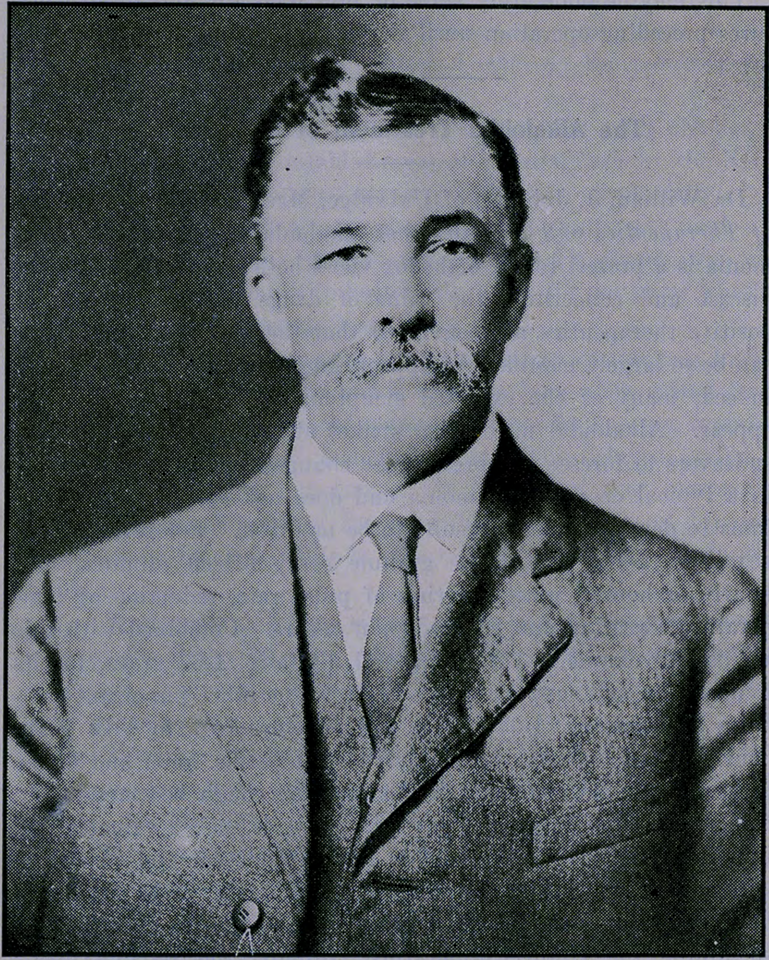

DR. ALEXANDER S. GARRETT,
Springtown, Texas.
Candidate for Congressman-at-Large from Texas.

Dr. Garrett announces his candidacy for Congressman-at-large, subject to action of the Democratic party. He stands for all that is desirable and needed by the medical profession and the people generally, for the promotion of sanitary science in the interest of the public health and race preservation; of just laws regarding labor and capital, whereby strikes may be avoided, a liability law for injuries in the industrial and transportation activities; government inspection of public works and supervision of the construction of all buildings with an eye to sanitation in both living and working structures; a National Department of Public Health. He is opposed to licensing the retailing of liquor by either State or general government; and is, therefore, a prohibitionist.

We need just such a man in Congress—able, fearless, earnest and conscientious. Dr. Garrett is one of the best known and foremost physicians of the State. He is a native of Georgia; M. D. from Atlanta Medical College, class of March, 1890. Has practiced medicine in Texas twenty-two years, and is 50 years of age.

